# Janus Distillation Membrane via Mussel-Inspired Inkjet Printing Modification for Anti-Oil Fouling Membrane Distillation

**DOI:** 10.3390/membranes13020191

**Published:** 2023-02-03

**Authors:** Morteza Afsari, Myoung Jun Park, Noel Jacob Kaleekkal, Mxolisi M. Motsa, Ho Kyong Shon, Leonard Tijing

**Affiliations:** 1 Centre for Technology in Water and Wastewater (CTWW), School of Civil and Environmental Engineering, University of Technology Sydney, 15 Broadway, P.O. Box 123, Ultimo, NSW 2007, Australia; 2 ARC Research Hub for Nutrients in a Circular Economy (NiCE), School of Civil and Environmental Engineering, University of Technology Sydney, 15 Broadway, P.O. Box 123, Ultimo, NSW 2007, Australia; 3 Membrane Separation Group, Department of Chemical Engineering, National Institute of Technology Calicut (NITC), Kozhikode 673601, India; 4 Institute for Nanotechnology and Water Sustainability (iNanoWS), College of Science, Engineering and Technology, University of South Africa, Johannesburg 1709, South Africa

**Keywords:** Inkjet printing, Janus membrane, asymmetric wettability, membrane distillation, oil fouling, surface modification, desalination

## Abstract

In this work, inkjet printing technology was used to print a thin layer of a hydrophilic solution containing polydopamine as a binder and polyethyleneimine as a strong hydrophilic agent on a commercial hydrophobic membrane to produce a Janus membrane for membrane distillation. The pristine and modified membranes were tested in a direct-contact membrane distillation system with mineral oil-containing feedwater. The results revealed that an integrated and homogenous hydrophilic layer was printed on the membrane with small intrusions in the pores. The membrane, which contained three layers of inkjet-printed hydrophilic layers, showed a high underwater oil contact angle and a low in-air water contact angle. One-layer inkjet printing was not robust enough, but the triple-layer coated modified membrane maintained its anti-oil fouling performance even for a feed solution containing 70 g/L NaCl and 0.01 v/v% mineral oil concentration with a flux of around 20 L/m^2^h. This study implies the high potential of the inkjet printing technique as a facile surface modification strategy to improve membrane performance.

## 1. Introduction

Water scarcity issues around the world have sparked innovations and developments in water desalination technologies to provide freshwater [1]. Reverse osmosis as the state-of-the-art membrane desalination technology is highly efficient; however, it is an energy-intensive process, and is salinity-limited in operation. Membrane distillation (MD), as an emerging hybrid thermal–membrane desalination technology that can utilize low grade or waste heat, has garnered increasing interest in recent years due to its ability to treat hypersaline solutions [2]. In MD, a hydrophobic membrane is used to separate a hot feed and a cool permeate, wherein the vapor pressure difference induced by the temperature gradient is the driving force. Some studies have also indicated the high potential of MD to treat challenging wastewaters containing surfactants and oil, such as those from coal seam or shale gas-produced water. However, the hydrophobic nature of the MDs membranes makes them susceptible to the attachment of hydrophobic fouling agents to the surface of the membrane [3]. The presence of some hydrocarbon pollutants in wastewater can cause hydrophobic–hydrophobic adherence, quick fouling, and wetting of the membrane [3,4,5]. Previous studies have shown that providing an asymmetric wettability on the membrane as a Janus structure, such as adding a thin hydrophilic layer on top of the hydrophobic membrane, could help alleviate the effect of oil fouling formation [4,6]. Various approaches have been carried out to provide a thin hydrophilic layer, such as vacuum filtration, layer-by-layer assembly, electrospinning, co-casting phase inversion, and electrospraying. These techniques have resulted in an improved performance; however, most of these processes face challenges in terms of non-uniform deposition, less precise coating, the difficulty of thickness control, and the consumption and wastage of materials. Thus, the fabrication of a homogenous and thin hydrophilic layer, which is strongly integrated into the support layer, is one of the main challenges in the fabrication of a Janus membrane. The intrusion of the hydrophilic layer into the channels of the microporous hydrophobic membrane can accelerate the wetting of the membrane [7]. To address these challenges, our approach uses an inkjet printing technique to modify a hydrophobic membrane and print a very thin hydrophilic layer, which provides a Janus property.

Inkjet printing is a commonly used printing method wherein small ink droplets are dropped onto a flat printing surface and they produce a thin and homogenous printed coating layer [8]. This technology has many advantages, such as being simple and fast, widely available, cheap in production, efficient in using materials, thereby limiting waste, and being a highly scalable technology [9,10]. In fact, inkjet printing has been used in many applications such as electronics, catalysts, biological cells, and sensors. The unique and simple working strategy of inkjet printing has received increasing interest toward the deposition of particles on the surface of materials. Zhang et al. have used inkjet printing to enhance the hydrophobicity of the surface of paper [11]. In recent years, inkjet printing has also found its way to application in the membrane technology field. For example, a study by Park et al. has inkjet-printed a thin layer of carbon nanotubes as an interlayer to enable a high-performance thin film composite nanofiltration membrane [12]. In another study, graphene oxide together with dopamine was used to coat a nanofiltration membrane via inkjet printing to add antifouling and chlorine resistance properties [13]. However, so far, there has been no study carried out on the modification of a hydrophobic membrane via inkjet printing for membrane distillation applications, especially dealing with oily feedwater.

In this study, the capability of inkjet printing to make an integrated, thin, and homogenous layer was used for the deposition of a thin layer of hydrophilic materials on the surface of a hydrophobic membrane to fabricate a Janus membrane with a high anti-oil fouling resistivity. We used dopamine as the hydrophilic polymer and binder on the surface of the membrane. Dopamine is a mussel-inspired material that has the ability to self-polymerize on both organic and inorganic surfaces without any damage to the surface, and it can provide functional groups for the grafting of other polymers or nanoparticles [14]. Therefore, polydopamine (PDA) acts as a powerful binding agent between a membrane and polyethyleneimine (PEI). PEI has the ability to bond with both primary and secondary amine groups. Therefore, it can covalently graft with quinone functional groups of dopamine and attach to the surface of the membrane. The other amine functional groups of the PEI can enhance the hydrophilic characteristics of the surface and make a hydrophilic layer [15]. The effect of the thickness of the coated layer was investigated, including the effect of the addition of a hydrophilic layer on one or both sides of the membrane. The anti-oil fouling performance for the membrane distillation was studied using feedwater containing different concentrations of mineral oil and NaCl solutions. The results demonstrated that the application of inkjet printing technology is highly interesting for the facile modification of membranes with robust properties for water treatment and desalination applications.

## 2. Materials and Methods

### 2.1. Materials

A commercial polyvinylidene (PVDF) membrane (Durapore®) with a pore size of 0.22 µm was purchased from Merck Millipore Ltd. (Burlington, MA, USA). Dopamine hydrochloride (MW = 189 g/mol), polyethylenimine (PEI) (MW = 2500), and tris(hydroxymethyl) aminomethane hydrochloride (Tris-HCl), which were used for polymerization of dopamine solution, were all bought from Sigma-Aldrich (Burlington, MA, USA). Sodium chloride (NaCl) and mineral oil were bought from local markets. All chemicals were used as received without any further purification. Deionized (DI) water was produced by a Millipore Milli-Q water system (Merck KGaA, Darmstadt, Germany). The printing procedure was performed using a portable Deskjet 2130 HP printer (Shanghai, China).

### 2.2. Inkjet Printing Modification

**Figure 1:**
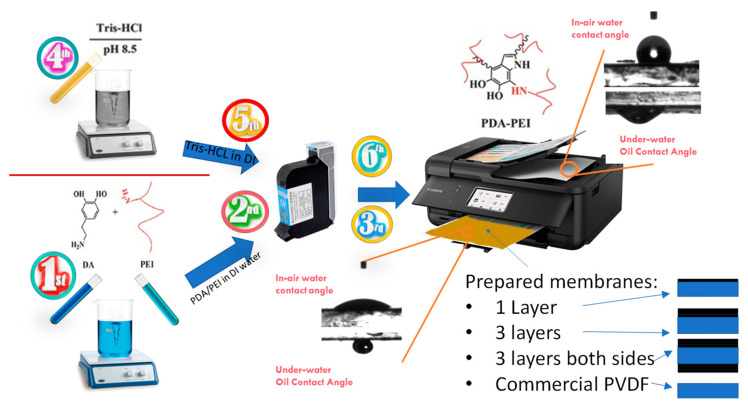
Schematic of the solution preparation and procedure for inkjet printing modification of a commercial hydrophobic membrane. The lower right figures show the schematic representation of the coating configurations of the prepared membranes.

Figure 1 shows a schematic representation of the solution preparation and inkjet-printing modification procedure carried out in this study. Briefly, two different solutions were first prepared: a dopamine/PEI solution and a Tris-HCl solution. In the first solution, 0.7 g dopamine and 0.7 g PEI were added to 50 mL DI water and stirred for 4 h at room temperature to make a homogenous solution of PDA/PEI. The color of the solution after mixing turned dark brown. Tris-HCl powder was poured into the DI water in another beaker to produce 50 mM Tris-HCl solution. The pH of the solution was adjusted to 8.5 by using 1 M HCl solution. Afterwards, a 4 cm × 6 cm black pattern was drawn in PowerPoint software and printed on A4-sized piece of paper to run the print order exactly on the membrane sample. Then, A4-sized PET film was placed on the printed paper and the commercial membrane was cut in 4 cm × 6 cm size and then taped on the PET film, accurately aligning with the previously printed pattern. Printing resolution was set to a maximum (1200 × 1200 dpi), with glossy paper as the paper type. Then, the prepared solutions were poured into clean and dried cartridges. In order to have homogenous printing, the printing order was placed multiple times to get high-quality print on the A4 paper. Then, the membrane taped on the PET film was loaded into the printer and printing order was placed to print the DA/PEI solution on the membrane. Afterwards, another cartridge containing Tris-HCl was loaded and printed on one side of the membrane to accelerate dopamine polymerization. This process was repeated three times on another membrane to have 1-time and 3-times coated membranes. For the fabrication of double-sided modified membranes, the printing procedure was performed three times on both sides of the membrane. Thus, three layers of DA/PEI and Tris-HCl were subsequently printed on one side, and after one day of drying the membrane at room temperature, the other side was taped on the PET film, and the printing procedure was performed three times on the other side of the membrane. Finally, there are four types of membrane labelled as 1-P (one side and one layer), 3-P (one side and three layers), D-P (double-side printed), and Com-PVDF (commercial PVDF membrane).

### 2.3. Membrane Characterization

The membrane surface was characterized using scanning electron microscopy (SEM, Zeiss Supra 55VP, Carl Zeiss AG, Jena, Germany). Furthermore, energy dispersive spectroscopy (EDS) mapping was taken using SEM instrument to analyze the distribution of elements on the membrane surface. The changes in wettability of the membranes surface were characterized by measurement of contact angle using Theta Kite 100, Biolin Scientific goniometer (Beijing, China) via a sessile drop method. The measurements and analysis were repeated at least three times to ensure their repeatability, and the average was reported [13].

The membrane surfaces of both commercial and modified membranes were characterized by attenuated total reflectance Fourier transform infrared (ATR-FTIR, Affinity-1 Shimadzu, Kawasaki, Japan) spectrometer to check the functional groups on the surface. These functional groups can determine the interaction of the fouling material with the membrane. Furthermore, the functional groups can ensure the completion of the polymerization of the dopamine as well as the interaction of the coated layer with the membrane surface.

The porosity of the membrane was measured using a gravimetric method. In this method, the membrane sample was dried, weighed, and then immersed in a wetting liquid. Then, the two sides of the membrane were gently wiped using an adsorbent tissue to remove excess wetting liquid. The change in weight of dry and wet samples was used for the measurement of the porosity of the membrane. The membrane porosity is calculated as follows [16]:

ε = ((W_1_ − W_2_)/D_e_)/([(W_1_ − W_2_)/D_e_] + W_2_/D_p_) (1)

where W_1_ and W_2_ are the wet and dry weights (g) of the samples, D_e_ and D_p_ are the density (g/m^3^) of the liquid and polymer, respectively.

The liquid entry pressure (LEP) determines the wetting resistivity of the hydrophobic membranes [17]. Higher LEP indicates good wetting resistance. LEP was measured using a lab-made setup composed of a cell filled with DI water, and the membrane sample was placed and tightened on top of the water. Then, water pressure was step-wise increased using compressed air. An increase in pressure causes the penetration of DI water into the pores of the membrane which can finally wet it. The minimum pressure that water passes through the membrane and water liquid, which is observed on the other side of the membrane, is the LEP of the membrane.

### 2.4. Membrane Distillation and Oil Fouling Tests

**Figure 2:**
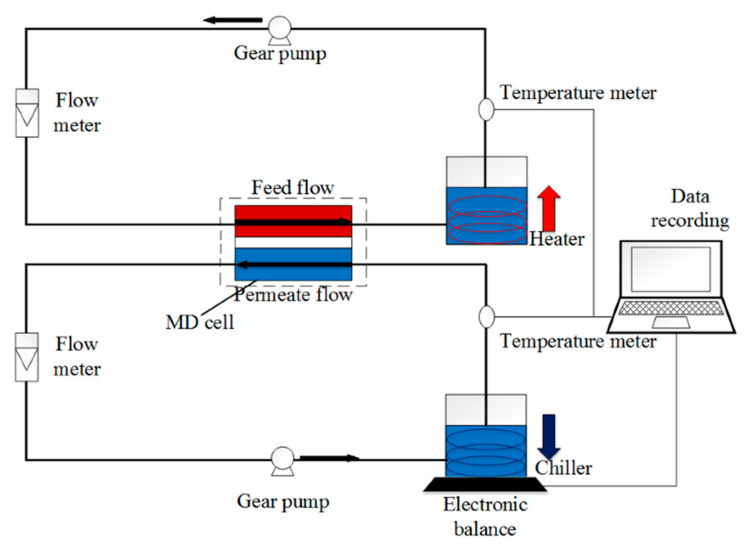
Schematic of the DCMD setup. Feed and permeate solutions at inlet temperatures of 60 and 20 °C, respectively, are flowing in counter-current mode at both sides of the hydrophobic membrane with controlled flow and temperature.

To determine both the fouling and wetting resistivity of the membranes, direct-contact membrane distillation (DCMD) test was performed in a lab-scale DCMD system similar to our previous study [18], as shown in Figure 2. In this system, the membrane sample, wherein the modified hydrophilic layer faces the feed solution, is placed in an MD module with an effective surface area of 9 cm^2^. The feedwater at 60 °C was passed through the feed side of the membrane, and on the other side, DI water that was cooled by a chiller and maintained at 20 °C was passed in a counterflow set-up. Feed and permeate flow rates were maintained at 400 and 350 cm^3^/min, respectively. The flow rate of the feed was set slightly higher than the permeate to increase the hydraulic pressure of the feed side and help the fast detection of wetting.

The performance of the membranes was evaluated by testing at different salinities (0, 35, 50, and 70 g/L) of the feed solution, with and without oil contaminant. During the MD tests, oil was introduced into the feedwater every 3 h, and the salt rejection and flux of the membranes were measured to evaluate the separation performance of the membranes. The hydrophobic PVDF membrane is oleophilic, has tendency to adsorb mineral oil, and showed potential for rapid fouling for the mineral oil. The oily feedwater was prepared by adding appropriate amounts of mineral oil into the NaCl solution and was vigorously stirred using a laboratory mixer at 2000 rpm for 30 min. The prepared oil concentrations in the feedwater were 0.001 v/v% (equivalent to 8 mg/L), 0.005 v/v% (40 mg/L), and 0.01 v/v% (80 mg/L). These values were selected to simulate the oil in a shale gas-produced water. For more homogenous mixing, the solution was heated to 50 °C and a 0.5 wt% of non-ionic surfactant (Tween 80) was added to produce an emulsion. The concentration of Tween 80 was optimized to make a homogenous emulsion. The concentration of the NaCl was adjusted to 0, 35, 50, and 70 g/L to simulate the effectiveness of the membranes in harsh conditions.

## 3. Results and Discussion

### 3.1. Morphology and Physical Characterization

**Figure 3:**
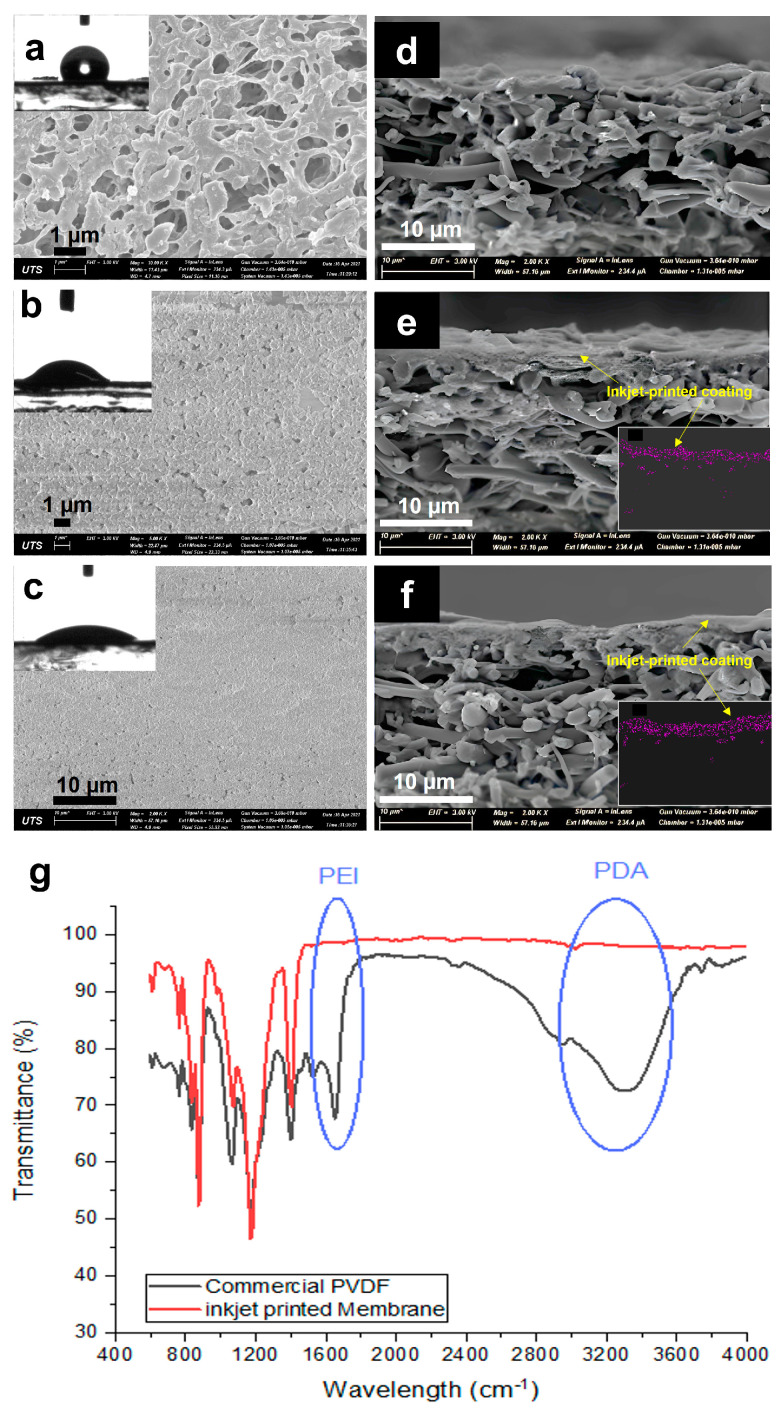
SEM images of the top (**a**–**c**) and cross-sectional (**d**–**f**) surfaces of the (**a**,**d**) commercial PVDF membrane, (**b**,**e**) 1-P membrane, and (**c**,**f**) 3-P membrane. (**g**) FTIR spectra of the commercial PVDF and inkjet-modified membrane (3-layers). Inset images of (**a**–**c**) show the corresponding water contact angles of the membranes, while inset images of e and f present the EDS mapping of Na on the coated hydrophilic layer of 1-P and 3-P membranes.

In order to investigate the effect of the modification process on the morphology of the membrane as well as on the change in the physical properties of the membranes, SEM images were taken and compared. Figure 3 shows the SEM images of the top surface and cross-section of the commercial and modified membranes and the corresponding FTIR spectra. The commercial membrane (Figure 3a) showed a highly microporous surface with interconnected pores. The cross-sectional image (Figure 3b) proves the symmetric structure of the membrane, which provides homogenous distribution of the porosity through the depth of the membrane. After inkjet modification, a homogenous layer was seen to cover the membrane surface and did not affect much of the bulk PVDF substrate. At the one-layer coating, the surface pores of the commercial membrane started to diminish, with only small pores left visible. It is indicated that the one-layer coating was insufficient for homogeneous coverage of the pristine membrane surface, with some parts showing defects. The cross-section showed an asymmetric structure that was composed of a thin hydrophilic coating and a thick hydrophobic pristine PVDF membrane. While the three-layer printing showed a much more uniform and homogenous coating over the surface of the membrane, with much fewer visible pores left. This indicates a better coating at a higher number of printing layers. The cross-sectional images (Figure 3e,f) confirm the presence of the printed layers on top of the membrane surface, with the three layers showing a bit of a thicker coating (~4 µm compared to 2 µm for one-layer). There is also a partial penetration of the top layer coating to the pristine base membrane, which provides a good anchor to the base membrane [19]. To further confirm the successful deposition of the printed coating layers on the membrane surface and to help estimate the layer thickness, the membranes used for the NaCl test were dried. EDS mapping was then taken to measure the amount of sodium on the membrane surface. Insets in Figure 3e,f show the presence of Na on the top hydrophilic layer, indicating that salt only stayed at the top hydrated layer and did not penetrate the bottom hydrophobic layer. This EDS map also gives an estimate of the thickness of the hydrophilic inkjet-printed layer.

Further confirming the successful printing of the hydrophilic layer is through the ATR-FTIR analyses of the membranes. Figure 3g shows new peaks for the inkjet-printed membrane (3-layers) in comparison to the unmodified commercial PVDF membrane at around 3300 cm^−1^, which is attributed to the presence of PDA relating to N−H and O−H stretching vibrations [20], verifying the polymerization of DA on the surface of the membrane. Furthermore, the peak at around 1648 cm^−1^ that corresponds to the stretching vibration of the C=N bonds [21] indicates the presence of PEI, which provides amine functional groups for hydrogen bonding and added hydrophilic property.

Table 1 shows the various physical characteristics of the commercial and modified membranes. As shown in the table, the overall porosity of the membrane showed only a slight decrease with the addition of very thin hydrophilic coating layers. The thickness of the hydrophilic layer is too thin that when compared to the bulk membrane thickness, it is just too small, as seen in the thickness measurement results. This result indicates that the negligible PDA-PEI hydrophilic thickness did not alter much of the membrane porosity.

### 3.2. Contact Angle (CA)

The water and oil wettability of the surface of commercial and modified membranes was analyzed by a sessile drop method to measure the contact angles. The insets of Figure 3 present the corresponding in-air water contact angles (WCA). The pristine PVDF membrane showed a WCA of 117°, indicating a hydrophobic surface. However, after the inkjet printing modification, the WCA of the 1-P membrane decreased to 34°, while for the 3-P membrane, it further reduced to 26°. Both printed membranes indicated hydrophilic surface modification, which confirms the presence of the successful coating of the hydrophilic layers. The better hydrophilicity of 3-P membrane could be due to a more uniform and homogenous coating along the surface of the membrane. This change in wettability enables the membrane to resist hydrophobic interaction with hydrophobic foulants such as oil [19].

We further tested the membrane for its in-air oil wettability, using a similar procedure for WCA measurement but using mineral oil. The commercial membrane showed quite in-air oleophilic behavior, continuously decreasing the CA of oil droplets to less than 10°, while the 1-P membrane showed an in-air OCA of 51° and revealed a higher repulsion than the commercial membrane. The in-air OCA for the 3-P membrane was even higher, almost reaching hydrophobic behavior (85°). However, for the membrane to have an anti-oil fouling effect, the membrane needs to have underwater oleophobicity. Thus, we tested our modified membranes for their oil contact angle underwater (last column in Table 1). The underwater OCA was measured by the immersion of membranes in DI water and the placement of oil drops on the membrane. The underwater OCA results showed that the oil droplet directly spread on the commercial membrane, showing high oleophilicity of the membrane in underwater condition. However, the inkjet-modified membranes showed a different behavior, with the underwater OCA for the 1-P and 3-P membranes reaching 93° and 152°, respectively. This result shows an acceptable oleophobicity of the modified membrane. This result is attributed to the formation of a hydration layer due to the presence of the thin hydrophilic layer. In other words, the hydrophilic characteristic of a coated layer increases the interaction of water and membrane and can prevent the attachment of oil droplets onto the membrane surface [22]. This interaction was derived due to the presence of hydrophilic functional groups such as amine, which provides strong hydrogen bonding with water droplets, which forms an interfacial hydration layer [23] and increases the oleophobicity of the modified surface, especially at an increasing thickness of the printed layer. The hydrogen bonding is strong and needs a high energy demand to destruct, whereas oil cannot afford this value of energy to adsorb on the surface and foul the membrane.

### 3.3. Liquid Entry Pressure (LEP)

LEP, which is the minimum transmembrane pressure needed for a liquid to penetrate the largest pore of the membrane, is an important parameter for the long-term performance of MD [24]. LEP is measured based on the Young–Laplace equation, which is a function of the geometric pore coefficient, the surface tension of the liquid, the wettability of the surface, and is inversely proportional to the maximum pore size. The LEP results (see Table 1) indicated that the commercial membrane had the highest LEP at 225 kPa, while the inkjet-modified membranes had slightly lower LEPs. This is interesting, as even though there was an addition of a hydrophilic layer, there was not much reduction in the LEP of the modified membrane in the range of 205–215 kPa. The LEP values for the 1-P and 3-P membranes were approximately similar. Other studies have also observed similar results where the addition of a thin hydrophilic layer barely influenced the LEP of the membrane [22]. The small reduction in LEP may be due to minimal penetration of the hydrophilic coating layer into the membrane, wherein the total length of the hydrophobic membrane base is slightly changed. However, for D-P, wherein the two sides have been inkjet print-coated, there must be an added penetration at the other side of the membrane where there is a bigger pore size; thus, this penetration has resulted in the lowering of the effective thickness of the pristine hydrophobic base membrane. In other words, the inkjet printing provided a homogenous and thin-thickness layer that prevented the high intrusion of the coating solution into the membrane’s pores and limited the decrease in the membrane’s LEP. As the inkjet printing did not really affect the pore size and porosity much (as presented in Table 1), the membranes still maintained their intrinsic characteristics, making them attractive for MD application.

### 3.4. DCMD and Anti-Oil Fouling Test

**Figure 4:**
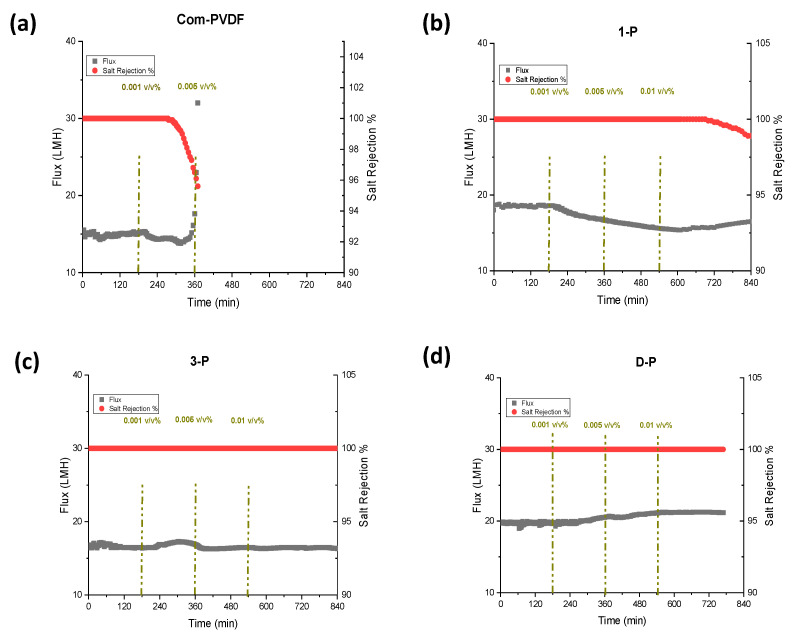
DCMD and anti-oil fouling performance showing flux and rejection for the commercial and the inkjet modified membranes: (**a**) Commercial PVDF membrane, (**b**) 1-P (1 layer inkjet coating), (**c**) 3-P (3-layer inkjet coating), and (**d**) D-P (double-sided 3-layer inkjet coating). The DCMD test was carried out at feed/permeate inlet temperatures of 60/20 °C and using 35 g/L NaCL solution with and without mineral oil addition of 0.001, 0.005, and 0.01 v/v% at 3 h interval.

Figure 4 presents the results for DCMD tests at a feed solution of 35 g/L NaCl and at feed and permeate inlet temperatures of 60 °C and 20 °C, respectively. During the tests, the first three hours were only the 35 g/L NaCl solution without the oil addition was used, after which various oil emulsion concentrations (0.001, 0.005, and 0.01 v/v% of oil) were added to the feed solution at 3 h intervals. The oil emulsion was prepared by first dispersing oil in water, and a small amount of Tween 80 as a surfactant was added and stirred using a high-speed mechanical mixer. From Figure 4a, the commercial membrane showed a constant flux of 15 LMH for the first 3 h, and then after adding 0.001 v/v% of mineral oil, the flux started to decrease, but the salt rejection remained stable and constant (100%). This indicated that the mineral oil has a high tendency to adhere to a hydrophobic membrane and gradually fouled the membrane covering the pores because of the long-range hydrophobic–hydrophobic interaction [25,26], thus declining the flux. However, the rejection still remained high for quite some time, and started to decrease after more than 2 h of additional operation. However, at a point where 0.005 v/v% was added, the flux started to increase, indicating that wetting had slowly occurred and that the rejection was further reduced. This could be attributed to the bridging effect, when some salts penetrate with the oil filling the pores, resulting in some water molecules bridging to the other side of the membrane to start wetting. This result shows a very weak performance of commercial membranes in oil-containing feedwater.

On the other hand, the test using the 1-P membrane showed a slightly higher flux at 18 LMH in the first few hours, which showed a small enhancement compared to a commercial PVDF membrane. This slight increase can be attributed to the potentially decreased thickness of the hydrophobic layer due to the small penetration of the hydrophilic coated layer that improved the mass transfer coefficient. The addition of 0.001 and 0.005 v/v% led to a gradual decrease in flux, indicating a partial fouling formation. The salt rejection in the first steps remained constant. Afterwards, at a 0.01 v/v% oil addition, the flux started to increase along with the decrease in salt rejection, indicating that the wetting of the membrane started to occur at this high oil concentration.

The 3-P membrane (Figure 4c) showed a lower flux than the 1-P membrane but was still higher than the commercial membrane. This could be due to the way the inkjet printing process for the second and third printing was conducted. In printing the first layer, the solution tends to intrude into the top part of the membrane and slightly decreases the bulk hydrophobic layer thickness, which could have caused an increase in the flux. After the completion of the polymerization step, the first layer plays as a barrier against the intrusion of the second- and third-layer’s printed solution onto the membrane. Therefore, further printing just makes a barrier against water transmission to the hydrophobic layer that can slightly decrease the flux. Even when the oil concentration was increased in the feed, the flux and salt rejection remained constant throughout the test, which indicated that there was no fouling or wetting that occurred during the test. This could be due to the interaction between the water molecules and the hydrophilic layer that maintains a hydration layer that prevents oil from attaching to the surface [27]. This makes the 3-P membrane an attractive membrane with oil-fouling resistivity in MD. With this positive enhancement of oil resistivity, we also prepared a membrane with 3-layer printing, but this time, at both sides of the membrane (D-P membrane). It is interesting to see from Figure 4d that the D-P membrane obtained the highest flux among the prepared membranes, at 20 LMH, which is 30% higher than the commercial membrane. This enhancement in the flux is attributed to the decrease in the hydrophobic membranes bulk’s thickness due to the slight penetration of the hydrophilic inkjet coating on both sides of the membrane, which reduces the mass transfer resistance and accelerates the condensation process. The presence of a hydrophilic layer on the permeate side does not have an influence on the anti-oil fouling performance of the membrane but can accelerate the condensation of the water vapor as well as help the mechanical stability of the hydrophobic membranes with lower thicknesses. The combination of an oil-barrier layer on the feed side and a hydrophilic layer on the permeate side enhanced the flux without sacrificing the salt rejection performance of the membrane. The membrane showed an approximately stable performance throughout the test with a complete salt rejection. This result implies that the use of inkjet printing as a coating technique is a good approach for uniform and homogenous coating of the hydrophilic layer to the membrane surface only, without compromising the hydrophobicity of the pristine PVDF base membrane material that maintains high rejection, and therefore improved the MD performance. This approach of providing both hydrophilicity and oleophobicity on the membrane, rather than just hydrophilicity for fouling control, has also been reported in the literature [22,28].

**Figure 5:**
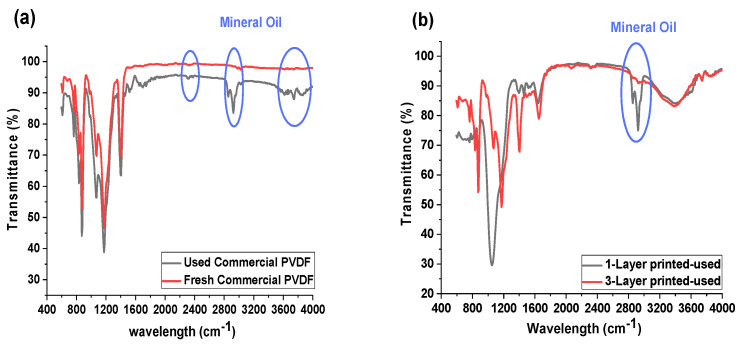
FTIR spectra of the various membranes: (**a**) commercial PVDF membrane before and after oil-fouling test, (**b**) 1-P and 3-P membrane after oil-fouling test.

Figure 5 shows the ATR-FTIR spectra of the commercial and inkjet-printed membranes before and after the oil-fouling test. FTIR analysis can be used for the determination of the attachment of fouling agents onto the membranes [18]. The FTIR results obtained several new peaks, which are representative for the mineral oil for the commercial membrane (Figure 5a) after the test. This confirms that oil has adhered to the commercial membrane surface. Similarly, for the 1-P membrane, the same peaks for mineral oil appeared on its FTIR data (Figure 5b), which also corroborates the result for the flux performance, with the decrease in the flux data for the 1-P membrane due to the oil fouling layer formation on the membrane. However, the membrane coated with a three-layer hydrophilic material (3-P) showed no mineral oil peaks and revealed a high membrane resistivity against fouling. This result proves the lower interaction between mineral oil and a hydrophobic membrane in a higher thickness of the hydrophilic layer that directed no fouling to the membrane, while the 1-P membrane still retains the mineral oil-hydrophobic interaction, which in the long term, caused fouling of the membrane.

**Figure 6:**
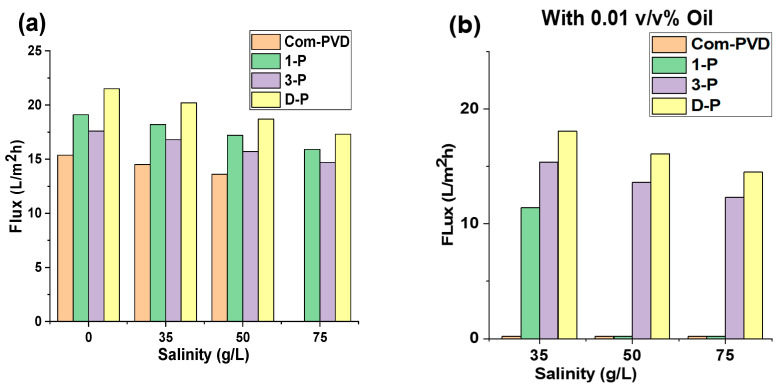
Effect of salinity on the performance of the different membranes in this study. Flux performance (**a**) without oil contaminant in feed, and (**b**) with 0.01 v/v% mineral oil in feed solution.

Furthermore, we also investigated the effect of salinity, with and without oil contaminant, on the DCMD performance of the unmodified commercial PVDF and the inkjet-modified Janus membranes. As shown in Figure 6a, the flux of all the membranes decreased with an increase in salinity. In general, the flux in the MD systems depends on the driving force provided by the difference in vapor pressure of the liquids on both sides of the membrane. According to the thermodynamic correlations, the vapor pressure of the liquids directly depends on the temperature. Therefore, the temperature difference between the two sides of the membrane makes a vapor pressure gradient and is the main source of the driving force of MD. However, the temperature difference is not the only influential parameter, and the driving force also depends on other factors, such as salinity. In fact, the vapor pressure of the feedwater is affected by the activity of the water on the surface, which is measured according to the molality of the feedwater [19]. The results prove that the flux of the membrane decreased by about 20% with an increment of the salinity from 0 to 75 g/L. The experiments took 4 hours for each step. The pristine membrane dealt with wetting in the last step (salinity of 75 g/L), mainly caused by the clogging of the pores and changes in the wettability of the membrane surface that caused the formation of the water channels on the membrane. However, the modified membrane showed better resistivity during the test and could retain its salt rejection throughout the DCMD test. The flux for D-P was higher than other membranes in different salinity ranges, but it dealt with the decrease in flux with the increase in salinity. In general, the salinity affected the vapor pressure of the feedwater and decreased the derived flux of the membranes.

We then tested the membranes at various feedwater salinities containing 0.01 v/v% of mineral oil (Figure 6b). Similar to the previous observation, the increase in salinity also led to a decrease in the driving force, resulting in a lower flux. In some cases, the presence of oil in the feedwater accelerated the pore blocking of the membranes. The commercial membrane had its pores blocked with oil, leading to a low flux. The 1-P membrane also was partially blocked after 8 h of the test, which was caused by mineral oil fouling. Although the 1-P membrane showed less than a 5% decrease in flux in the feedwater with no oil, the oily saline water dramatically decreased the flux of the 1-P membrane. In this case, the flux showed a 40% decline compared to the initial flux. Afterwards, the membrane started to sharply decrease in flux and was blocked. However, one-layer coating caused more tolerability compared to the commercial membrane. The 3-P and D-P membranes could retain their complete salt rejection performance even in an oily and high salinity feedwater, but their flux was stepwise decreased by an average of a 30% flux decline compared to their initial fluxes. The results show that the decline in flux for oily feedwater is higher than for the no-oil feedwater. The average flux decline in each step for the membranes was less than 7%, with a total flux decrease of less than 20% for the DCMD test for feedwater containing no oil. These values for the treatment of feedwater containing 0.01 v/v% (or equivalent to 80 mg/L) mineral oil were more than 10% for each step and were about 30% for the total period of the test. The higher decrease in flux for oily feedwater can be attributed to the changes in the vapor pressure of the feedwater. The presence of any impurity can affect the vapor pressure of the water. The boiling point of mineral oil is higher than 300 °C, and the dispersed oil can decrease the evaporation rate of water and consequently, decrease the vapor pressure and the driving force of the MD process [21,22].

## 4. Conclusions

In this study, we have tested the feasibility of using inkjet printing as a coating approach to producing a Janus membrane with a commercial PVDF membrane as a pristine base membrane for membrane distillation application. The printing process was performed on a commercial membrane to print one to three layers of a hydrophilic solution containing DA and PEI. Then, a Tris-HCl solution was printed to complete the dopamine polymerization and make an integrated hydrophilic layer. The effect of the printing layer thickness on its desalination and anti-oil fouling performance was investigated. The FTIR spectra proved the completion of the polymerization of dopamine, and the oil/water contact angle data revealed a change in the wettability of the membrane after the modification process. The membranes were tested to analyze their antifouling resistivity, and the experiments showed that commercial membranes showed a high susceptibility to mineral oil fouling, while the Janus membrane could tolerate fouling. The thicker (three layers) hydrophilic layers provided higher fouling resistivity, even at oil concentrations up to 0.01 v/v%. The 3-P and D-P membranes showed the highest fluxes (17–20 LMH) and maintained a high rejection rate (>99.9%) compared to the commercial and 1-P membranes. This study signified the potential of a facile inkjet printing coating method to produce a Janus membrane that can withstand oil fouling for membrane distillation. This opens up further exploration of such an approach for the preparation of functional membranes that are suitable for challenging wastewater treatment and desalination.

## Figures and Tables

**Table 1:** Physical characteristics of the inkjet-modified and commercial membranes.

Sample	LEP (kPa)	Membrane Thickness (µm)	Porosity (%)	WCA (deg)	In-Air OCA (deg)	Underwater OCA (deg)
C-PVDF	225	112 ± 1.40	75 ± 0.86	117	10	0
1-P	215	113 ± 1.23	73 ± 0.65	34	51	93
3-P	214	114 ± 1.55	73 ± 0.91	26	85	152
D-P	205	115 ± 1.85	72 ± 0.74	26	85	152

LEP: liquid entry pressure; WCA: in-air water contact angle; OCA: oil contact angle.

## Data Availability

Data available on request.
